# Gender Difference in the Association Between Subjective Socioeconomic Mobility Across Life Course and Mortality at Older Ages: Results From the JAGES Longitudinal Study

**DOI:** 10.2188/jea.JE20190083

**Published:** 2020-11-05

**Authors:** Yuiko Nagamine, Takeo Fujiwara, Yukako Tani, Hiroshi Murayama, Takahiro Tabuchi, Katsunori Kondo, Ichiro Kawachi

**Affiliations:** 1 Center for Preventive Medical Science, Chiba University, Chiba, Japan; 2 Department of Global Health Promotion, Tokyo Medical and Dental University, Tokyo, Japan; 3 Department of Family Medicine, Tokyo Medical and Dental University, Tokyo, Japan; 4 Institute of Gerontology, The University of Tokyo, Tokyo, Japan; 5 Cancer Control Center, Osaka International Cancer Institute, Osaka, Japan; 6 Department of Gerontological Evaluation, Center for Gerontology and Social Science, Aichi, Japan; 7 Center for Well-being and Society, Nihon Fukushi University, Aichi, Japan; 8 Department of Social and Behavioral Sciences, Harvard T.H. Chan School of Public Health, Boston, MA, USA

**Keywords:** subjective socioeconomic status, trajectory, all-cause mortality, gender roles, Japan

## Abstract

**Background:**

Socioeconomic mobility affects health throughout the life course. However, it is not known whether there are gender differences in the association between life-course subjective socioeconomic status (SSS) mobility and mortality at older ages.

**Methods:**

Participants were 16,690 community-dwelling adults aged 65–100 years in the Japan Gerontological Evaluation Study (JAGES). Baseline information including demographic characteristics, depression, and lifestyle factors were collected in 2010. Participants’ vital status was confirmed in 2013 via linkage to death records. We categorized life-course socioeconomic mobility into the following categories: ‘persistently high’, ‘downward mobility’, ‘upward mobility’, and ‘persistently low’. Cox proportional hazard modeling was used to estimate hazard ratios (HR) for all-cause mortality.

**Results:**

Mortality HRs for the ‘downward’ group were 1.37 (95% confidence interval [CI], 1.08–1.74) among men and 1.27 (95% CI, 0.94–1.71) among women in comparison with the ‘persistently high’ group. Compared to the ‘persistently low’ group, the HRs for the ‘upward’ group were 0.54 (95% CI, 0.35–0.83) among women and 0.91 (95% CI, 0.73–1.24) among men. Associations were not changed after adjusting for objective socioeconomic status but were attenuated by depression.

**Conclusions:**

‘Downward’ mobility was associated with mortality among men, but not among women. Depression appeared to mediate the association. A protective effect of upward mobility was observed among women but not among men.

## INTRODUCTION

Subjective socioeconomic status (SSS), defined as “a person’s belief about his location in a status order”,^1^ has been shown to be correlated with health and illness independently from objective socioeconomic status (SES; including educational attainment, income, and occupation).^2^ Singh-Manoux et al concluded that SSS can capture a perceived average of objective SES.^3^ More recently, the biological pathways linking SSS to health have begun to be documented. People’s perceptions of their social status predict health behaviors, such as smoking^4^ and sedentarism,^2^ as well as some biomarkers, such as heart rate, sleep latency, cortisol habituation to stress, body fat distribution,^5^ and high waist circumference.^6^ Additionally, SSS has been shown to be inversely related to metabolic syndrome,^6^ insulin resistance,^7^ coronary artery diseases, hypertension, diabetes and dyslipidemia.^8^ As for older people, difficulties in instrumental activities of daily living are also inversely related to SSS.^2^ Presumably, through these pathways, low SSS is associated with excess risk of mortality.^9^ The statistically significant associations between SSS and all outcomes above—except coronary artery disease, hypertension, and diabetes—were observed even after adjusting for objective socioeconomic status.

Recent studies have begun to focus on the trajectories of SSS across the life course. For example, Burazeri et al^10^ found that downward mobility in SSS (ie, high SSS in childhood/low SSS during mid-life) was associated with increased risk of acute coronary syndrome (ACS) for both men and women at around 60 years of age. In this study, financial loss appeared to mediate downward mobility and ACS. Depressive symptoms were not considered in the study, although depression might be a robust mediator of the association between SSS and ACS. Another study of Latino and Asian immigrants in the United States (US) found that downward subjective social mobility was associated with elevated risk of major depression.^11^^,^^12^ However, to the best of our knowledge, no studies have examined the association between mobility in SSS and mortality among older people prospectively, independent from objective SES, and the mediating effect of depression in this association.

It is also not known whether there are gender differences in the association between mobility in SSS and health outcomes. Previous studies on “objective” social class mobility have found gender differences. For example, Cambois et al examined occupational class mobility and standardized mortality rates (SMR) stratified by sex in a French population aged 30–84 years old.^13^ In this study, occupational class was classified into six groups: ‘upper classes’, ‘craft, trade, etc’, ‘farmers’, ‘clerks’, ‘manual workers’ and ‘inactive’. Among both men and women, individuals who were downwardly mobile had statistically significant higher mortality compared to ‘non-movers’. At the same time, men who moved upwards from ‘inactive’ to a higher occupational position also experienced higher mortality risk compared to non-movers. By contrast, among women, moving up from the ‘inactive’ group showed a protective effect on mortality risk.^13^ A study in Poland reported that upward intergenerational educational mobility (comparing individuals to their fathers) was protectively associated with cardiovascular risk—as measured by the Framingham risk score (FRS)—only among men.^14^ No study has investigated whether there are gender differences in the association between “subjective” social class mobility and mortality.

Gender roles closely mirror the cultural context. For example, in Japan’s traditional patriarchal culture, women can be college-educated, but still end up as home-makers and, therefore, do not benefit from upward educational mobility to the same extent as men.^15^ We derive two theoretical predictions for the gender difference we should expect to observe in the association between mobility in SSS and mortality. In a rigidly traditional society (men are expected to be bread-winners and women are expected to stay at home), (i) upward social mobility may not be beneficial to women; and (ii) in the same society, downward mobility could be more “toxic” for men because the male is the breadwinner and the household’s fortunes are more closely linked to the male’s SES trajectory. In this present study, these hypotheses were examined.

The Japan Gerontological Evaluation Study (JAGES) is a large-scale, population-based cohort study established in 2010 that collected data on both childhood and current SSS among healthy older (65 years or older) community residents in Japan. Vital status was ascertained in the cohort via linkage to death registry data. Previously, using prospective JAGES data from 2003, we reported that lower objective SES (educational attainment and household income) was associated with higher mortality.^16^ However, the impact of differential trajectories of SSS on mortality risk has not been investigated in older Japanese adults. In the present study, our objectives were to examine whether trajectories of SSS across the life course (ie, differences in subjective social status mobility from childhood to old age) are associated with the risk of mortality and whether there is a gender-related difference in the association.

## METHODS

### Study participants

Data from an ongoing prospective longitudinal cohort study, the Japan Gerontological Evaluation Study (JAGES), were analyzed. The cohort was established with the aim of elucidating the determinants of healthy ageing among community-dwelling seniors. The baseline survey was administered between August 2010 and January 2012, when survey questionnaires were mailed to 131,468 community-dwelling individuals aged ≥65 years who were physically and cognitively independent. People from 24 municipalities throughout Japan were invited to participate. Participants were randomly selected from the official residence registries in 13 large municipalities; and in the remaining 11 smaller municipalities, census data of all eligible residents was sought. A total of 86,063 subjects returned the questionnaire (response rate: 65.5%).

Approximately one-fifth of the total sample (*N* = 19,528) was randomly selected to receive a supplementary survey module inquiring about their childhood and current SSS. After excluding those who had missing SSS responses (*N* = 2,832) as well as individuals who could not be linked to mortality register data (*n* = 6), our analytical sample consisted of 16,690 subjects (7,944 men and 8,746 women). The JAGES protocol was approved by the Ethics Committee in Research of Human Subjects at Nihon Fukushi University (No. 10-05) and the Ethics Committee of Chiba University, Faculty of Medicine (No. 1777).

### Ascertainment of death

Participants were matched to death records from the public long-term care insurance system, which is maintained by local municipal authorities throughout Japan. All deaths were reported and submitted by physicians to municipalities. In the analysis sample from 2013, 780 deaths were found, which was 4.7% of total participants at baseline. Of these deaths, 524 were men (6.6% of 7,944 male participants), and 256 were women (2.9% of 8,746 female participants).

### Childhood and current SSS

Childhood SSS was asked using the following question: “How would you rate your social status when you were aged 15 years compared to others in society at that time?”.^17^ Possible responses were arranged on a 5-point Likert scale: ‘high’, ‘middle-high’, ‘middle’, ‘middle-low’, or ‘low’.

Current SSS was assessed via the question: “How would you rate your current social status in comparison with others in society?”, with the same 5-point Likert scale of responses.

Since a previous study showed that even subtle downward/upward mobility can be associated with health outcomes,^13^ we subtracted the 5-point Likert scale response for childhood SSS from the same 5-point Likert scale response for adult SSS, and categorized the resulting numbers into ‘downward SSS trajectory’ if the calculated result was −4 to −1, ‘no change’ if 0 and ‘upward SSS trajectory’ if +1 to +4. Subsequently, ‘no change’ was dichotomized into two groups and the individual was defined as ‘persistently high’ if both childhood and current SSS were ‘high’, ‘middle-high’ or ‘middle’, and ‘persistently low’ if both SSS were ‘middle-low’ or ‘low’.

### Covariates

Self-reported adult height, current income, education, and marital status were included as potential confounding factors. Additionally, depression and lifestyle factors were included as potential mediators of the association between SSS mobility and mortality. Adult height was used as a proxy of childhood adversity, such as childhood nutrition and disease status.^18^^,^^19^ Adult height was categorized into five groups in 5-cm intervals specific to each gender: for men: <155, 155–159.9, 160–164.9, 165–169.9, and ≥170 cm; and for women: <145, 145–149.9, 150–154.9, 155–159.9, and ≥160 cm.^17^ Education was assessed as years of schooling (0–9, 10–12, or ≥13 years), and annual household income was equalized by dividing the gross income by the square root of the number of household members (<2.00, 2.00–3.99, or ≥4.00 million yen). We decided not to include occupational class as a covariate after performing the sensitivity analysis. Marital status was categorized as married, widowed, divorced, unmarried, or others. The 15-item short form of the Geriatric Depression Scale (GDS) (Japanese version) was used to assess depression and categorized as no depression (0–4), moderate depression (5–9), and depression (10+).^20^^,^^21^ Lifestyle factors included smoking status (current smoker/ex-smoker or non-smoker), alcohol intake (current drinker/ex-drinker or non-drinker), walking time per day (<30 min or ≥30 min), and body mass index (BMI) (underweight [<18.5 kg/m^2^], normal [18.5–24.9 kg/m^2^], overweight [25.0–29.9 kg/m^2^], or obesity [≥30.0 kg/m^2^]).^22^

### Statistical analysis

Data was analyzed by gender because self-rated health^23^ and depressive symptoms^24^^,^^25^ differed between men and women in our sample.^26^^,^^27^ Cox proportional hazards models were used to estimate hazard ratios (HRs) and 95% confidence intervals (CIs) for all-cause mortality during the 3-year follow-up period. The following sequential multivariable-adjusted models were constructed: model 1 was adjusted for age; model 2 was additionally adjusted for adult height, adult objective SES (educational and income level), and marital status; model 3 was additionally adjusted for depression; and model 4 was additionally adjusted for lifestyle factors. Analysis was performed using Stata/SE version 13.1 (Stata Corp, College Station, TX, USA).

## RESULTS

The overall and gender-stratified characteristics of the study subjects are described in Table 1. Among all participants, 25.7% of men and 31.5% of women reported both a high childhood SSS and high current SSS, while 18.7% of men and 14.3% of women reported low current SSS and childhood SSS. Twenty percent of men and 30.0% of women were categorized as having ‘downward mobility’, while 35.6% of men and 24.2% of women were categorized as having ‘upward mobility’ (Table 1).

**Table 1.** Characteristics of participants

	Men (*n* = 7,944)	Women (*n* = 8,746)
	*N* (%)	*N* (%)
Age, years		
65–69	2,731 (34.4%)	2,844 (32.5%)
70–74	2,332 (29.4%)	2,531 (28.9%)
75–79	1,656 (20.8%)	1,800 (20.6%)
≥80	1,225 (15.4%)	1,571 (18.0%)
Mobility of SSS		
Persistently high	2,042 (25.7%)	2,756 (31.5%)
Downward mobility	1,588 (20.0%)	2,622 (30.0%)
Upward mobility	2,829 (35.6%)	2,114 (24.2%)
Persistently low	1,485 (18.7%)	1,254 (14.3%)
Height^a^		
Short	580 (7.3%)	1,071 (12.2%)
Middle-short	1,331 (16.8%)	2,442 (27.9%)
Middle	2,498 (31.4%)	2,884 (33.0%)
Middle-tall	2,066 (26.0%)	1,461 (16.7%)
Tall	1,092 (13.7%)	389 (4.4%)
Missing	377 (4.7%)	499 (5.7%)
Equalized income, million yen		
High (≥4.00)	851 (10.7%)	782 (8.9%)
Middle (2.00–3.99)	2,846 (35.8%)	2,569 (29.4%)
Low (<2.00)	3,201 (40.3%)	3,506 (40.1%)
Missing	1,046 (13.2%)	1,889 (21.6%)
Education, years		
High (≥13)	1,654 (20.8%)	971 (11.1%)
Middle (10–12)	2,528 (31.8%)	2,930 (33.5%)
Low (≤9)	3,465 (43.6%)	4,538 (51.9%)
Other/Missing	297 (3.7%)	307 (3.5%)
Marital status		
Married	6,748 (84.9%)	5,056 (57.8%)
Widowed	611 (7.7%)	2,846 (32.5%)
Divorced	181 (2.3%)	321 (3.7%)
Unmarried	102 (1.3%)	190 (2.2%)
Others/Missing	302 (3.8%)	333 (3.8%)
Smoking status		
Non-smoker	1,873 (23.6%)	7,066 (80.8%)
Smoker/ex-smoker	5,516 (69.4%)	674 (7.7%)
Missing	555 (7.0%)	1,006 (11.5%)
Alcohol intake		
Current drinker/Ex-drinker	4,778 (60.1%)	1,332 (15.2%)
Non-drinker	2,698 (34.0%)	6,923 (79.2%)
Missing	468 (5.9%)	491 (5.6%)
Walking time		
<30 min/day	2,354 (29.6%)	2,869 (32.8%)
≥30 min/day	5,156 (64.9%)	5,279 (60.4%)
Missing	434 (5.5%)	598 (6.8%)
Body weight status (BMI), kg/m^2^		
Underweight (<18.5)	424 (5.3%)	699 (8.0%)
Normal (18.5–24.9)	5,397 (67.9%)	5,749 (65.7%)
Overweight (25.0–29.9)	1,612 (20.3%)	1,550 (17.7%)
Obesity (≥30.0)	128 (1.6%)	228 (2.6%)
Missing	383 (4.8%)	520 (5.9%)
Depressive symptoms		
No depression (GDS <5)	4,970 (62.6%)	5,130 (58.7%)
Moderate depression (GDS 5–9)	1,406 (17.7%)	1,490 (17.0%)
Depression (GDS ≥10)	496 (6.2%)	539 (6.2%)
Missing	1,072 (13.5%)	1,587 (18.1%)

BMI, body mass index; GDS, Geriatric Depression Scale; SSS, subjective socioeconomic status.

^a^Height (<155, 155–159.9, 160–164.9, 165–169.9, and ≥170 cm for men and <145, 145–149.9, 150–154.9, 155–159.9, and ≥160 cm for women).

Among men, after adjustment for age, the HR from the ‘downward mobility’ group was 1.37 (95% CI, 1.08–1.74) compared to those in the ‘persistently high’ group (model 1, Table 2). Even after adjustment for adult height, adult objective SES, and marital status, the HR of ‘downward mobility’ was significantly higher compared to ‘persistently high’ (model 2, HR 1.35; 95% CI, 1.05–1.72). Additional adjustment for depression attenuated the association between ‘downward mobility’ and mortality, which became non-significant (model 3, HR 1.22; 95% CI, 0.95–1.56). Additional adjustment for lifestyle factors showed a non-significant association (model 4, HR 1.22; 95% CI, 0.95–1.56). On the other hand, compared to the ‘persistently low’ group, no significant association was found between ‘upward mobility’ and mortality among men. The relationships mentioned above were not changed even after adjustment for area and the longest job (eTable 1, eTable 3).

**Table 2.** Hazard ratio of SSS mobility, SES, and other covariates among men (*N* = 7,944)

	Model 1	Model 2	Model 3	Model 4
Persistently high	ref	ref	ref	ref
Downward	**1.37 (1.08–1.74)**	**1.35 (1.05–1.72)**	1.22 (0.95–1.56)	1.22 (0.95–1.56)
Upward	0.87 (0.69–1.09)	0.85 (0.68–1.08)	0.83 (0.66–1.04)	0.84 (0.66–1.05)
Persistently low	0.95 (0.73–1.24)	0.91 (0.69–1.20)	0.83 (0.63–1.10)	0.78 (0.59–1.03)
	
	Model 5	Model 6	Model 7	Model 8
	
Persistently high	1.05 (0.80–1.37)	1.09 (0.83–1.44)	1.20 (0.91–1.58)	1.28 (0.97–1.69)
Downward	1.44 (1.10–1.88)	1.47 (1.13–1.93)	1.46 (1.12–1.91)	1.56 (1.19–2.04)
Upward	0.91 (0.71–1.18)	0.94 (0.72–1.22)	0.99 (0.76–1.29)	1.07 (0.82–1.39)
Persistently low	ref	ref	ref	ref

BMI, body mass index; GDS, Geriatric Depression Scale; SES, socioeconomic status; SSS, subjective socioeconomic status.

Models 1 and 5 were adjusted for age.

Models 2 and 6 were additionally adjusted for height, equivalised income, education, marital status.

Models 3 and 7 were additionally adjusted for GDS.

Models 4 and 8 were additionally adjusted for smoking status, drinking habits, BMI and walking times.

Interestingly, the trend was opposite in women (Table 3). Women from the downward mobility group showed no significant excess mortality risk compared to the ‘persistently high’ group either before (model 1, HR 1.27; 95% CI, 0.94–1.71) or after adjustment for age, adult height, objective SES, and other covariates (model 4, HR 1.08; 95% CI, 0.79–1.47). Although the result was not statistically significant, the point estimate of the HR showed that adjustment for depression considerably attenuated the effect of downward mobility among women also. We additionally conducted a check for the interaction between gender by mobility in the SSS and mortality. The result confirmed that downward mobility was harmful only among men. However, compared to the ‘persistently low’ group, the age-adjusted HR of the ‘upward mobility’ group was 0.54 (95% CI, 0.35–0.83; model 5). After adjustment for adult height, adult objective SES, and marital status, the HR for ‘upward mobility’ was slightly attenuated to 0.58 (95% CI, 0.38–0.90; model 6). In the final model, after additional adjustment for depression and other covariates, the HR for ‘upward mobility’ was attenuated to statistical non-significance (model 8, HR 0.68; 95% CI, 0.44–1.07). Also, among women, the relationships were not amended by the adjustment for area and the longest job (eTable 2 and eTable 4).

**Table 3.** Hazard ratios of SSS mobility, SES, and other covariates among women (*N* = 8,746)

	Model 1	Model 2	Model 3	Model 4
Persistently high	ref	ref	ref	ref
Downward	1.27 (0.94–1.71)	1.22 (0.90–1.65)	1.10 (0.81–1.50)	1.08 (0.79–1.47)
Upward	0.71 (0.49–1.04)	0.69 (0.48–1.02)	0.68 (0.47–1.00)	0.69 (0.47–1.01)
Persistently low	1.33 (0.91–1.94)	1.20 (0.81–1.76)	1.07 (0.72–1.58)	1.01 (0.68–1.50)
	
	Model 5	Model 6	Model 7	Model 8
	
Persistently high	0.75 (0.52–1.10)	0.83 (0.57–1.23)	0.94 (0.63–1.38)	0.99 (0.67–1.47)
Downward	0.95 (0.66–1.38)	1.02 (0.70–1.48)	1.03 (0.71–1.49)	1.06 (0.73–1.55)
Upward	**0.54 (0.35–0.83)**	**0.58 (0.38–0.90)**	**0.64 (0.41–0.99)**	0.68 (0.44–1.07)
Persistently low	ref	ref	ref	ref

BMI, body mass index; GDS, Geriatric Depression Scale; SES, socioeconomic status; SSS, subjective socioeconomic status.

Models 1 and 5 were adjusted for age.

Models 2 and 6 were additionally adjusted for height, equivalised income, education, marital status.

Models 3 and 7 were additionally adjusted for GDS.

Models 4 and 8 were additionally adjusted for smoking status, drinking habits, BMI and walking times.

## DISCUSSION

In this analysis of life-course trajectories of SSS among Japanese older adults, we found four notable patterns: (a) ‘downward mobility’ increased the risk of mortality compared to the ‘persistently high’ group only among men; (b) ‘upward mobility’ decreased the risk of mortality only among women, with no effect seen among men; (c) depression appeared to mediate the association between mobility in SSS and mortality; and finally, (d) objective SES in later life did not fully explain the relationship between mobility in SSS and mortality.

Our finding of excess mortality among downwardly mobile groups is consistent with previous studies in other populations and other age groups.^10^^–^^12^ However, it was observed only among men in the older Japanese population. Downward mobility increases the risk of poor health for men because of a subjective sense of deprivation in adult life relative to their childhood status. This might be less salient for women in Japanese society because their economic fortunes are more tightly linked to their spouse’s status. As shown in Table 1, over 90% of women in the sample were married or previously married (now widowed). Also, as mentioned above, the gender difference here is probably real because the formal test of interaction was statistically significant only among men. In addition to that, depression is probably mediating the association between downward mobility and mortality. Depression had the strongest attenuation effect among covariates in model 3, both among men and women.

Generally, upward SES mobility has a protective effect on health compared to persistently low SES.^28^ In our study, ‘upward’ SSS mobility attenuated mortality risks among women, but not among men. This pattern is contrary to our original hypothesis that upward mobility would not affect women’s risk, because even if women graduated from college, this would not be reflected by higher earnings/income. Many Japanese women of the generation in our sample became housewives irrespective of their years and quality of education.^15^ For men, we did not find either a harmful or protective effect of ‘upward’ mobility in this study. We can cite two possibilities why upward mobility is sometimes harmful. First, status incongruity refers to when upward mobility creates stress because the individual feels like an outsider in her new status/position. Striving for upward mobility and success can also induce stress, also known as the John Henryism phenomenon.^29^ Second, the “long arm” of low childhood SES may continue to exert an influence on health even if the individual manages to escape from lower status (ie, poor childhood SES persists even if someone achieves high SES in later life).^30^ For women in this study, there was a protective effect of ‘upward’ mobility. In theory, upward mobility for Japanese women could be even more stressful than in the west, because it was so non-normative and, consequently, women in positions of authority and power could become very isolated. However, in the age-group represented by our cohort, we may assume that the majority of ‘upward’ mobility more closely reflected their father’s or spouse’s social class rather than their own,^31^ so it may not be indicative of exposure to status incongruity.

Our results are also notable for finding that mobility in SSS had a similar impact on mortality, even after adjusting for objective SES. The contribution of SSS to mental and physical health—over and above the contribution of objective SES—has been previously discussed.^5^^,^^32^^,^^33^ Singh-Manoux et al investigated the determinants of SSS in a cohort of middle-aged British civil servants.^3^ They concluded that classic objective SES, such as occupational grade, income, and education, were highly correlated to SSS, but none of them fully explained the impact of SSS on health. Two possible explanations for the residual effect of SSS have been proposed^25^: (a) SSS may reflect an individual’s SES more comprehensively than objective SES by capturing, for example, feelings of financial security, stocks of wealth, the respect of peers, satisfaction with standard of living, and so on; and (b) SSS may capture not only the objective social grade, but also people’s sense of their relative position in society and its impact on health.

On the other hand, the possibility of reverse causation between SSS and health, especially mental health, has also been raised as an alternative explanation.^25^^,^^34^ That is, depressed individuals are more likely to make pessimistic evaluations of their past and present circumstances. However, a strength of our study is that the outcome was objective (mortality) and not self-assessed (such as depression), thereby avoiding the threat of common method bias.

Limitations of this study should be mentioned. First, childhood SSS was assessed via subjective recall. Previous research, however, supports the reliability of retrospective assessments of childhood SSS using siblings’ recall of childhood SSS.^35^ Second, our analysis was limited to all-cause mortality, and cause-specific mortality was not considered. Future studies should examine cause-specific mortality, such as coronary heart disease, stroke, or cancer, to clarify the effect of mobility in SSS among older Japanese adults, who are ranked as having the longest life expectancy in the world.^36^ Third, our findings may have been biased by selective survival due to the fact that all the participants were aged 65–100 years at baseline (ie, more vulnerable population may have already died before the start of follow-up). Last, the duration of follow-up (3 years) was comparatively short.

In conclusion, our study found a possible harmful effect of downward SSS mobility on mortality among Japanese older men and protective effect of upward SSS mobility among women. The adverse downward effect was mediated by depression. Additionally, mobility in SSS in later life may have higher predictive value on mortality related to depression than objective SES. Mobility in SSS from childhood to older age may be important to be considered when assessing the mortality risk of older people.

## APPENDIX A. SUPPLEMENTARY DATA

eTable 1. Hazard ratio of SSS mobility, SES, and other covariates among men (area adjusted) (N = 7,944)

eTable 2. Hazard ratios of SSS mobility, SES, and other covariates among women (area adjusted) (N = 8,746)

eTable 3. Hazard ratio of SSS mobility, SES, and other covariates among men (the longest job adjusted) (N = 7,944)

eTable 4. Hazard ratios of SSS mobility, SES, and other covariates among women (the longest job adjusted) (N = 8,746)
