# Supplementary material for: Gender Difference in the Association Between Subjective Socioeconomic Mobility Across Life Course and Mortality at Older Ages: Results From the JAGES Longitudinal Study
Source: J Epidemiol. 2020 Nov 5;30(11):497–502. doi: 10.2188/jea.JE20190083 (PMC7557171; doi:10.2188/jea.JE20190083)
Supplement: Supplementary file 1 [file je-30-497-s001.pdf]

eTable 1. Hazard ratio of SSS mobility, SES, and other covariates among men (area adjusted) (N=7,944)

	Model 1	Model 2	Model 3	Model 4
Persistently high	ref	ref	ref	Ref
Downward	**1.33 (1.05 - 1.69)**	**1.30 (1.02 - 1.66)**	1.20 (0.94 - 1.54)	1.22 (0.95 - 1.57)
Upward	0.90 (0.72 - 1.13)	0.89 (0.70 - 1.12)	0.86 (0.68 - 1.09)	0.88 (0.70 - 1.11)
Persistently low	0.96 (0.74 - 1.25)	0.93 (0.71 - 1.22)	0.86 (0.65 - 1.13)	0.84 (0.63 - 1.10)
	Model 5	Model 6	Model 7	Model 8
Persistently high	1.04 (0.80 - 1.36)	1.08 (0.82 - 1.42)	1.16 (0.88 - 1.54)	1.19 (0.91 - 1.58)
Downward	1.38 (1.06 - 1.81)	1.40 (1.07 - 1.84)	1.40 (1.07 - 1.83)	1.46 (1.11 - 1.91)
Upward	0.94 (0.73 - 1.21)	0.96 (0.74 - 1.24)	1.00 (0.77 - 1.31)	1.05 (0.81 - 1.37)
Persistently low	ref	ref	ref	Ref

BMI, body mass index; GDS, Geriatric Depression Scale; SES, socioeconomic status; SSS, subjective socioeconomic status.

Models 1 and 5 were adjusted for age and prefecture code

Models 2 and 6 were additionally adjusted for height, equivalised income, education, marital status

Models 3 and 7 were additionally adjusted for GDS***

Models 4 and 8 were additionally adjusted for smoking status, drinking habits, BMI**** and walking times

eTable 2. Hazard ratios of SSS mobility, SES, and other covariates among women (area adjusted) (N=8,746)

	Model 1	Model 2	Model 3	Model 4
Persistently high	ref	ref	ref	Ref
Downward	1.28 (0.95–1.72)	1.24 (0.91–1.68)	1.12 (0.82–1.52)	1.07 (0.78–1.45)
Upward	0.76 (0.52–1.10)	0.74 (0.51–1.08)	0.73 (0.50–1.07)	0.73 (0.50–1.07)
Persistently low	1.34 (0.92–1.96)	1.21 (0.83–1.79)	1.08 (0.73–1.60)	1.03 (0.69–1.53)
	Model 5	Model 6	Model 7	Model 8
Persistently high	0.75 (0.51–1.09)	0.82 (0.56–1.21)	0.92 (0.62–1.37)	0.97 (0.66–1.44)
Downward	0.95 (0.66–1.38)	1.02 (0.70–1.48)	1.03 (0.71–1.50)	1.04 (0.71–1.51)
Upward	**0.56 (0.37–0.87)**	**0.61 (0.39–0.94)**	0.67 (0.43–1.05)	0.71 (0.45–1.10)
Persistently low	ref	ref	ref	ref

BMI, body mass index; GDS, Geriatric Depression Scale; SES, socioeconomic status; SSS, subjective socioeconomic status.

Models 1 and 5 were adjusted for age and prefecture code

Models 2 and 6 were additionally adjusted for height, equivalised income, education, marital status

Models 3 and 7 were additionally adjusted for GDS***

Models 4 and 8 were additionally adjusted for smoking status, drinking habits, BMI**** and walking times

eTable 3. Hazard ratio of SSS mobility, SES, and other covariates among men (the longest job adjusted) (N=7,944)

	Model 1	Model 2	Model 3	Model 4
Persistently high	ref	ref	ref	Ref
Downward	**1.37 (1.08–1.74)**	**1.32 (1.03–1.69)**	1.19 (0.93–1.53)	1.18 (0.92–1.52)
Upward	0.87 (0.69–1.09)	0.86 (0.68–1.08)	0.83 (0.66–1.05)	0.84 (0.67–1.06)
Persistently low	0.95 (0.73–1.24)	0.90 (0.68–1.18)	0.82 (0.62–1.08)	0.76 (0.58–1.01)
	Model5	Model6	Model7	Model8
Persistently high	1.05 (0.80–1.37)	1.12 (0.85–1.47)	1.22 (0.92–1.61)	1.31 (0.99–1.74)
Downward	1.44 (1.10–1.88)	1.47 (1.12–1.93)	1.45 (1.11–1.90)	1.55 (1.18–2.03)
Upward	0.91 (0.71–1.18)	0.96 (0.74–1.24)	1.01 (0.78–1.32)	1.11 (0.85–1.44)
Persistently low	ref	ref	ref	Ref

BMI, body mass index; GDS, Geriatric Depression Scale; SES, socioeconomic status; SSS, subjective socioeconomic status.

Models 1 and 5 were adjusted for age and prefecture code

Models 2 and 6 were additionally adjusted for height, equivalised income, education, marital status

Models 3 and 7 were additionally adjusted for GDS***

Models 4 and 8 were additionally adjusted for smoking status, drinking habits, BMI**** and walking times

eTable 4. Hazard ratios of SSS mobility, SES, and other covariates among women (the longest job adjusted) (N=8,746)

	Model 1	Model 2	Model 3	Model 4
Persistently high	ref	ref	ref	Ref
Downward	1.27 (0.94–1.71)	1.20 (0.88–1.63)	1.09 (0.80–1.48)	1.06 (0.78–1.45)
Upward	0.71 (0.49–1.04)	0.71 (0.48–1.03)	0.70 (0.48–1.02)	0.71 (0.48–1.03)
Persistently low	1.33 (0.91–1.94)	1.20 (0.82–1.77)	1.08 (0.73–1.60)	1.02 (0.69–1.52)
	Model5	Model6	Model7	Model8
Persistently high	0.75 (0.52–1.10)	0.83 (0.56–1.22)	0.93 (0.63–1.37)	0.98 (0.66–1.45)
Downward	0.95 (0.66–1.38)	1.00 (0.69–1.45)	1.01 (0.70–1.47)	1.04 (0.71–1.51)
Upward	**0.54 (0.35–0.83)**	**0.59 (0.38–0.91)**	0.64 (0.42–1.00)	0.69 (0.44–1.08)
Persistently low	ref	ref	ref	ref

BMI, body mass index; GDS, Geriatric Depression Scale; SES, socioeconomic status; SSS, subjective socioeconomic status.

Models 1 and 5 were adjusted for age and prefecture code

Models 2 and 6 were additionally adjusted for height, equivalised income, education, marital status

Models 3 and 7 were additionally adjusted for GDS***

Models 4 and 8 were additionally adjusted for smoking status, drinking habits, BMI**** and walking times
